# Therapeutic efficacy of ranitidine bismuth citrate with clarithromycin for seven days in the eradication of *Helicobacter pylori* in Brazilian peptic ulcer patients

**DOI:** 10.1590/S1516-31802003000100004

**Published:** 2003-01-02

**Authors:** Jaime Natan Eisig, Fernando Marcuz Silva, Cláudio Hashimoto, Ethel Zimberg Chehter, Antonio Atilio Laudanna

**Keywords:** *Helicobacter pylori*, Eradication, Peptic ulcer treatment, Ranitidine-bismuth, Clarithromycin therapeutic use, *Helicobacter pylori*, Erradicação, Úlcera péptica tratamento, Bismuto

## Abstract

**CONTEXT:**

The curative treatment of peptic ulcer is made available nowadays through the eradication of the bacterium Helicobacter pylori, which is associated with it, but the best therapeutic regimen is yet to be determined.

**OBJECTIVE:**

To assess the efficacy of a therapeutic regimen with 400 mg ranitidine bismuth citrate associated with 500 mg clarithromycin given twice a day for seven days in a cohort of Brazilian patients with peptic ulcer.

**TYPE OF STUDY:**

Cross-sectional study.

**SETTING:**

Tertiary-care hospital.

**PATIENTS:**

One hundred and twenty nine outpatients, with active or healed peptic ulcers infected by Helicobacter pylori, diagnosed via endoscopy with confirmation via the urease test and histological examination, who had never undergone a regimen for the eradication of the bacterium.

**PROCEDURE:**

Administration of 400 mg ranitidine-bismuth and 500 mg clarithromycin twice a day, for seven days.

**MAIN MEASUREMENTS:**

Efficacy of the treatment, with a check on the cure done via another endos-copy eight weeks after drug administration. The eradication of the bacterium was determined via the urease test and histological examination. Patients who were negative for both were considered to be cured.

**RESULTS:**

Eight patients failed to complete the study. The eradication rate according to intention to treat was 81% (104/129) and per protocol was 86% (104/121).

**CONCLUSION:**

The bismuth ranitidine compound associated with clarithromycin used for one week was shown to be a simple, effective and well-tolerated therapeutic regimen for the eradication of *Helicobacter pylori*.

## RESUMO

**CONTEXTO:**

O tratamento curativo da úlcera péptica é proporcionado atualmente pela erradicação da bactéria *Helicobacter pylori*, a ela associada, mas o melhor esquema de erradicação ainda não está determinado.

**OBJETIVO:**

Avaliar a eficácia de um esquema terapêutico com citrato de bismuto ranitidina 400 mg associado a claritromicina 500 mg, ministrados duas vezes ao dia por sete dias em uma população de ulcerosos brasileiros.

**TIPO DE ESTUDO:**

Estudo transversal.

**LOCAL:**

hospital de atenção terciária.

**PARTICIPANTES:**

129 pacientes ambulatoriais, portadores de úlcera péptica ativa ou cicatrizada, diagnosticados por endoscopia e com infecção pelo *Helicobacter pylori* confirmada pelo teste da urease e exame histológico, que nunca foram submetidos a tratamento para erradicação da bactéria.

**PROCEDIMENTO:**

Administração dos medicamentos citrato de bismuto ranitidina 400 mg e claritromicina 500 mg duas vezes ao dia, durante sete dias.

**VARIÁVEIS ESTUDADAS:**

Eficácia de tratamento com o controle de cura em nova endoscopia realizada oito semanas após o uso dos medicamentos. A erradicação da bactéria foi deter-minada pelo teste da urease e por histologia, sendo considerados curados os pacientes com resultados negativos para os dois exames.

**RESULTADOS:**

Oito pacientes não completaram o estudo. O índice de erradicação por intenção de tratamento foi de 81% (104/129) e por protocolo, 86% (104/121).

**CONCLUSÃO:**

O composto bismuto-ranitidina, associado à claritromicina, utilizado por uma semana, mostrou ser um esquema bastante simples, eficaz e bem tolerado para a erradicação do *Helicobacter pylori*.

## INTRODUCTION

Since *Helicobacter pylori (H. pylori)* was discovered in the stomach in 1983 by Marshall and Warren,^1^ the natural history of peptic ulcers has undergone a radical change. The relationship between the presence of this bacterium and gastritis, gastric ulcer, duodenal ulcer^2^ and gastric cancer^3^ has been broadly accepted since then. Several studies have demonstrated that the eradication of the bacterium prevents ulcer recurrence.^4,5^

Different triple and quadruple therapeutic regimens, with a series of combinations using bismuth, anti-secretory drugs and antibiotics, have demonstrated great efficacy in the eradication of the bacterium.^6,7^ However, they present the problem of low compliance by the patient, plus the high incidence of side effects. The double regimens are better tolerated, but their efficacy is lower when compared with triple and quadruple regimens.^8,9^

Ranitidine bismuth citrate (RBC) shows protective activity on the mucous membrane, and anti-pepsin and anti-bacterial activity through the bismuth plus the anti-secretory action of ranitidine. Clarithromycin is a macrolide that has achieved eradication rates of up to 96%^10,11^ (per protocol) when administrated at the dose of 250 mg, four times a day in association with 400 mg RBC twice a day for 14 days.

With the objective of providing the most effective, best-tolerated and simplest therapeutic regimen for Brazilian peptic ulcer patients, with consequent high compliance, we assessed the efficacy of an *H. pylori* eradication regimen with RBC 400 mg twice a day in association with 500 mg clarithromycin twice a day for only one week.

## METHODS

Outpatients aged between 15 and 80 years, with duodenal or gastric ulcer diagnosed by upper digestive system endoscopy in the A, H or S phases, according to the Sakita classification criteria,^12^ were invited to participate in the study. Their *H. pylori* infection was confirmed by two diagnostic methods, the urease test and histological analysis, performed on biopsy material from the gastric antrum. In the urease test, in which Christensen's method was used (with urea as the substrate in a liquid medium and phenol red as the indicator), a mucosal fragment from the antrum was immersed and kept under observation for up to 24 hours. Cases were considered positive when there was a change in the pH indicator. In the histological examination, a mucosal fragment from the antrum stained with hematoxylin/eosin was analyzed by an expert pathologist. Cases were considered positive when the bacteria was identified, regardless of the density of bacterial colonization.

Patients with reflux esophagitis, complicated ulcer, previous gastric surgery, subchronic anti-inflammatory drug use, patients with severe illness, and pregnant or nursing women were excluded from the study. Patients who had previously undergone treatment for the eradication of *H. pylori* were also excluded from the protocol. The study was approved by the Ethics and Scientific Committee of our hospital and all patients signed an informed consent form.

All patients received 400 mg RBC and 500 mg clarithromycin twice a day. for seven days, in the morning under fasting conditions and at night, with an empty stomach. The patients were informed about the occurrence of dark stools caused by the use of bismuth as well as the possible side effects. At the end of the treatment, patients returned to the outpatient clinic for assessment of adverse effects and the counting of the remaining tablets. The adverse effects were evaluated in relation to the type of occurrence, its duration, the cause/effect relationship with the assessed medication, and the intensity. The latter was classified as slight, when the effect was easily tolerated; moderate, when causing discomfort without impairing daily activities; and severe, when the patient was forced to discontinue daily activities.

Eight weeks after treatment completion, the patients underwent another endoscopy with biopsy. The *H. pylori* infection was considered to have been cured when the urease test and histological examination proved negative.

***Statistical analysis***. The calculation of the sample size was determined by means of the descriptive study of a dichotomous variable, in which the prevalence of peptic ulcer in the general population was considered to be 10%, with an expected eradication efficacy of 90%. The eradication rates were calculated via intention to treat and per protocol analysis.

All patients enrolled in the study were taken to have the intention to treat. Since all of the patients enrolled took more than 80% of the medication, those who returned and agreed to undergo the control endoscopy were considered for the per-protocol analysis. The confidence interval of 95% was calculated for the eradication rates. The Chi-squared method was used for comparison of eradication rates according to risk factors, gender and ulcer type, with a significance value of p < 0.05.

Statistical calculations were performed using the statistics software Statistical Package, version 8.0 (SPSS Inc., USA).

## RESULTS

The demographic data for the population studied is shown in Table 1. The average age was similar to the median. The incidence of duodenal ulcer was higher than gastric ulcer incidence.

**Table 1:** Demographic data of patients enrolled in the study

Data		Values
Number. of patients		129
Age (years)	– Average	52
	– Median	52
	– Range	15-80
Male / Female (%)		51 / 49
Duodenal ulcer (%)		66
Gastric ulcer (%)		19
Duodenal and gastric ulcer (%)		15
Tobacco users (%)		26
Alcohol consumption (%)		2
Tobacco and alcohol users (%)		5

Of the 129 patients who received eradication treatment, 8 (6.2%) did not return for follow-up. The eradication rate for intention to treat was 81% and 86% per protocol (Table 2).

**Table 2:** Eradication of H. pylori rates

	n / %	Confidence Interval (95%)
"Per protocol"	104/121 ***(86)***	80% - 92%
Intention to treat	104/129 ***(81)***	74% - 88%

Risk factors for ulcers such as cigarette smoking and alcohol consumption, ulcer type and gender did not influence the therapeutic response (Table 3).

**Table 3:** Eradication of H. pylori rates and clinical features

Clinical Features	Eradication (%)	Value of p	
Gender	Female	81	0.13
	Male	91	
Type of ulcer	Duodenal	85	0.83
	Gastric	89	
	Gastric and duodenal	89	
Risk factors	Absent	89	0.52
	Cigarette smoking	93	
	Alcohol consumption	50	
	Cigarettes and alcohol	80	

Adverse effects were reported by 29% of the patients; 19% reported slight intensity, 8% moderate intensity and 2% severe intensity, but none of the patients had to discontinue treatment. All of the patients took more than 80% of the medication. The most frequent adverse effects are shown in Table 4.

**Table 4:** Adverse effects of the therapeutic regimen

Symptoms	Intensity (number of events)		
	Slight	Moderate	Severe
Bitter taste	6	5	1
Nausea	5	4	1
Diarrhea	6	1	1
Epigastric pain	5	1	
Headaches	1		
Anal pruritus	1		

## DISCUSSION

Several studies have demonstrated high efficacy and good tolerance of a double therapeutic regimen consisting of 400 mg RBC + 500 mg clarithromycin twice a day for 14 days, with *H. pylori* eradication rates of 71-83% (for intention to treat), and 81-96% (per protocol).^12-15^ Controversy still exists with regard to the duration of different treatment schemes for bacterial eradication, although studies have demonstrated an absence of significant difference for treatments that last 7, 10 or 14 days.^16,17^ European and Asiatic multicenter studies have considered a 7-day scheme to be effective.^18,19^

In Brazil, there is high prevalence of *H. pylori*, high incidence of strains resistant to nitromidazole and large segments of the population with low financial status. Thus, shorter and less expensive schemes with better compliance, using clarithromycin, are desirable. In our study, we obtained an eradication rate of 86% (per protocol) and 81% (per intention to treat), thereby achieving better compliance by the patient. There were fewer side effects, even with medication being administrated for one week.

The efficacy of this simple and well-tolerated therapeutic regimen is comparable to other triple or quadruple regimens that included bismuth for 7 to 14 days.^6,7^ The regimen had better results than for other double regimens, such as omeprazole plus clarithromycin.^20^ This efficacy is also comparable to many other triple schemes that did not use bismuth.^21^ In our service, a scheme consisting of omeprazol, clarithromycin and tinidazole given twice a day for 7 days, to patients who had not been treated previously, presented an eradication rate of 76%.^22^ A scheme of pantoprazole, clarithromycin and metronidazole, also given twice a day for 7 days, presented an eradication rate of 87% (in press). In a series of 411 patients treated with omeprazol, clarithromycin and amoxicillin for 7 days, we found eradication rates of 85% (per protocol) and 80% for intention to treat (data not published).

The treatment was well tolerated by the patients. The most common adverse effects were bitter taste in the mouth and diarrhea, but none of the patients had to discontinue treatment.

## CONCLUSION

The dual therapy with RBC 400 mg and clarithromycin 500 mg administered twice a day for only one week to treat Brazilian patients with peptic ulcer disease is a simple, well-tolerated and highly effective treatment for the eradication of *H. pylori*.
